# Design, Synthesis, and Antimicrobial Evaluation of New Annelated Pyrimido[2,1-*c*][1,2,4]triazolo[3,4-*f*][1,2,4]triazines

**DOI:** 10.3390/molecules25061339

**Published:** 2020-03-15

**Authors:** Islam H. El Azab, Nadia A.A. Elkanzi

**Affiliations:** 1 Chemistry Department, Faculty of Science, Taif University, Al-Haweiah, P.O. Box 888, Taif 21974, Saudi Arabia; 2 On leave from Chemistry Department, Faculty of Science, Aswan University, Aswan P.O. Box 81528, Egypt; kanzi20@yahoo.com; 3 Chemistry Department, College of Science, Jouf University, P.O. Box 2014, Sakaka, Saudi Arabia

**Keywords:** Mannich reaction, 1,2,4-triazine, pyrimidine, 1,2,4-triazole, *N*-heterocycles, antimicrobial activity

## Abstract

A series of 34 new pyrimido[2,1-*c*][1,2,4]triazine-3,4-diones were synthesized and fully characterized using IR, NMR, MS, and microanalytical analysis. In vitro investigation of 12 compounds of this series revealed promising antimicrobial activity of the conjugates **15a** and **15f**–**j** that were tagged with electron-withdrawing groups, with sensitivities ranging from 77% to as high as 100% of the positive control. The investigation of antimicrobial activity included *Bacillus subtilis* ATCC 6633, *Staphylococcus aureus* ATCC 6535, *Pseudomonas aeruginosa* ATCC 27853, and *Escherichia coli* ATCC 8739 (EC), and fungal strains *Candida albicans* ATCC 10231 and *Aspergillus brasiliensis* ATCC 16404.

## 1. Introduction

Genetic mutations are major contributors to the prognosis of drug-resistant microbial strains [1]. These strains are, in most cases, able to detoxify drugs using mutant digestive enzymes like β-lactamases [2]. In addition, they are able to prevent the intracellular build-up of drugs to microbially nontoxic levels using mutant drug efflux proteins [3]. Spontaneous, error-prone replication bypass, errors introduced during DNA repair, and induced mutations are the four main modes of mutation encountered in nature. Induced mutations, in particular, emerge after a gene has come into contact with a mutagen or environmental inducer [4].

Therefore, seeking alternatives to commercially available drugs that will, sooner or later, no longer be effective remains a pharmacological challenge. The discovery of new antibiotics and innovative pharmacophore architectures for synthesis in particular, those based on computer-aided drug design (CADD) programs [5], in addition to molecular library approaches provide opportunities to develop new drug candidates [6].

**Figure 1:**
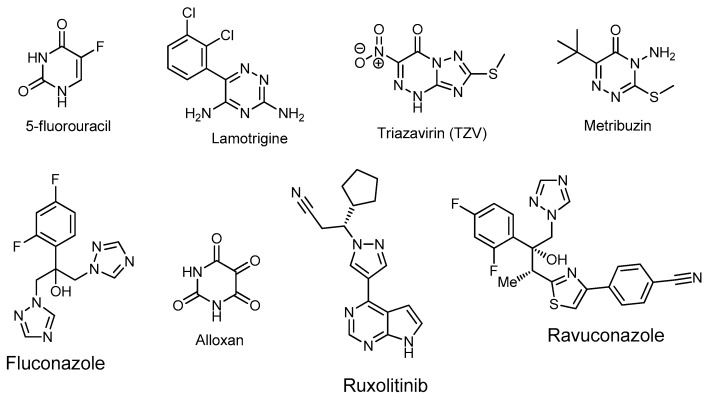
Representative examples of bioactive 1,2,4-triazole, 1,2,4-triazine, and pyrimidine derivatives.

Aza-heterocycles are amongst the medications which have diverse uses in the market (Figure 1). Of this class, 1,2,4-triazines [7,8,9,10,11,12,13] and 1,2,4-triazoles [14,15,16,17,18,19,20,21], as well as pyrimidines [22,23], have been introduced in a renewed generation of medication. Due to our interest in these azines, their ligation, and modification, we revisited and continued upon our previous research projects [24,25,26,27,28,29,30] with this study. We describe the annulation and in vitro preliminary antimicrobial impact of new 8-phenyl-6-(thiophen-2-yl)-6,7-dihydro-2*H*-pyrimido[2,1-*c*][1,2,4]triazine-3,4-dione tripod-based congeners.

## 2. Results and Discussion

### 2.1. Chemistry

**Scheme 1:**
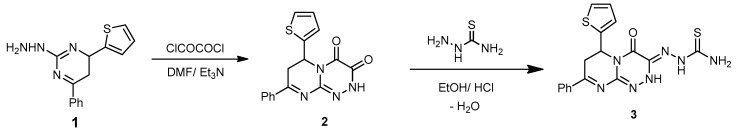
Reagents and conditions for synthesis of compounds (**2** and **3**).

As a part of our ongoing research toward the synthesis of a variety of nitrogen bridgehead heterocycles, we report the utility of the hydrazine derivative **1** [25,26] to construct fused pyrimidotriazine **2**, (Scheme 1). Treatment of the cyclic 1,2-bioxygen analogue **2** with thiosemicarbazide produced the thioureido analogue **3**, as mainly indicated by mass and/or NMR analyses. The presence of the thiocarbonyl group was deduced by ^13^C-NMR as a singlet at 181.2 ppm and its IR absorption band at 1290 cm^−1^, whereas the presence of the amino group was confirmed by ^1^H-NMR as a singlet at 8.56 ppm. The recorded mass at *m*/*z* 397.08 corresponds to the formula C_17_H_15_N_7_OS_2_. All these data support the formation of the thiosemicarbazone derivative **3** via simple condensation of one amino group to afford the thioureido analogue **3** without further cyclocondensation.

**Scheme 2:**
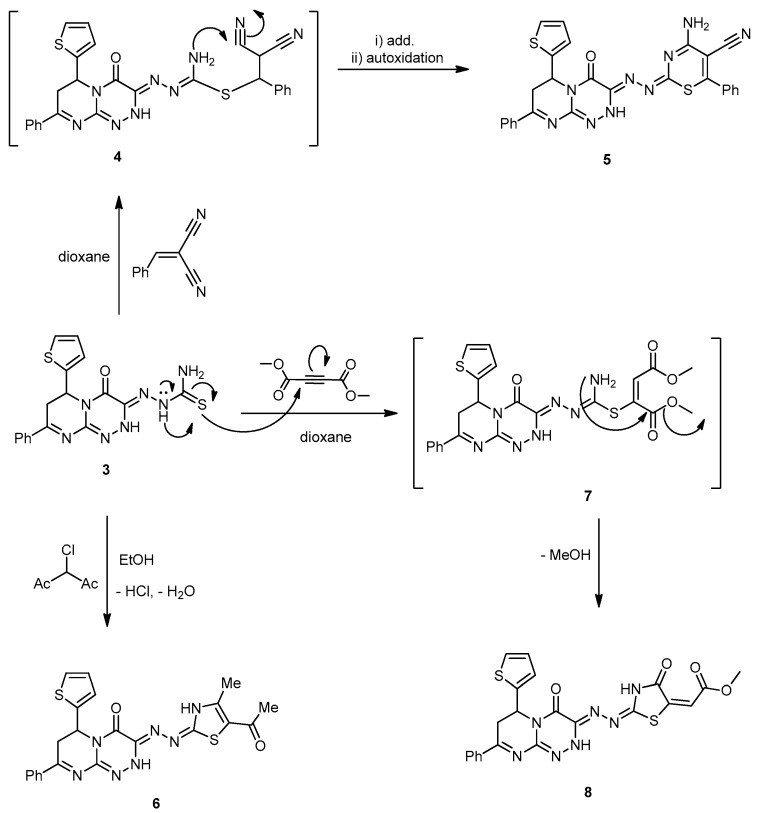
Reagents and conditions for the synthesis of compounds **5**, **6**, and **8.**

The thioureido analogue **3** was subject to a sequence of treatments to investigate the reactivity of its thioureido moiety in an attempt to attain target thiazole and/or thiazine architectures. Thus, the treatment of compound **3** with benzylidenemalononitrile in boiling dioxane led to 1,3-thiazine-5-carbonitrile derivative **5** via the carbonitrile intermediate 4. This intermediate undergoes intramolecular cyclization via the nucleophilic addition of NH_2_ to the nitrile group, affording the 1,3-thiazine analogue **5** with a 75% yield (Scheme 2). Strong absorption bands at 3325 and 2219 cm^−1^ were observed for the NH_2_ and C≡N groups, respectively. Upon treatment of the thioureido **3** with 3-chloropentane-2,4-dione in refluxing EtOH, the 2-substituted 4-methyl-5-acetylthiazole derivative **6** was obtained. The most characteristic signal of compound **6 (**^1^H-NMR), due to the thiazole exchangeable (N–H) proton at 10.83 ppm, in addition to two new singlets, were observed at 2.51 and 2.72 ppm, attributed to the methyl and acetyl protons, respectively. Taken together, these data confirmed the structure of compound **6**.

Similarly, compound **3** was cyclized with dimethyl but-2-ynedioate in refluxing dioxane to annulate the thiazole analogue **8** with an 80% yield (Scheme 2). Formation of compound **8** can be explained on the basis of an initial Michael-type addition of the thiol function in the thioureido moiety to the activated triple bond in dimethyl but-2-ynedioate to afford the non-isolable intermediate **7**, which undergoes intramolecular cyclization via loss of another MeOH molecule (route a) to yield the thiazole derivative **8**. The carbothioamide absorption bands originally observed in **3** at 1290 and 3230 cm^−1^ disappeared after this reaction.

**Scheme 3:**
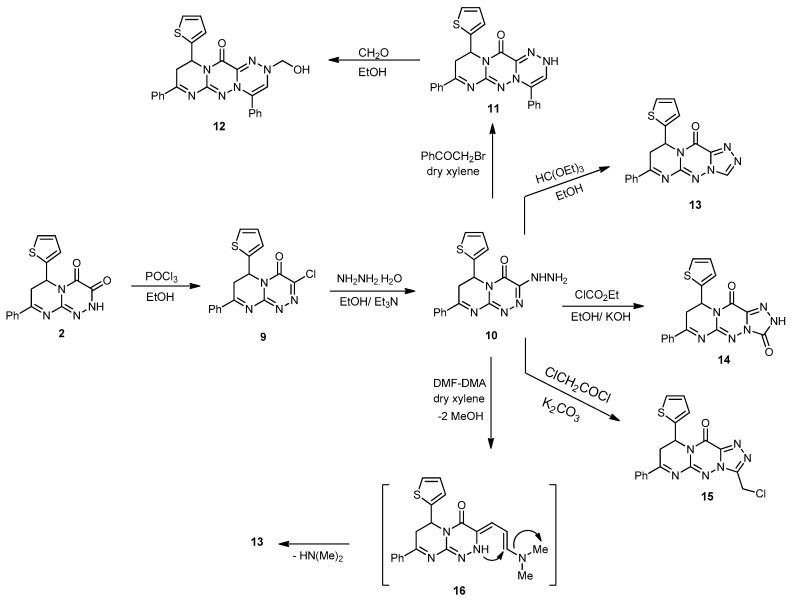
Reagents and conditions for the synthesis of compounds **9**–**15**.

Chlorination of the 1,2-dioxo compound **2** with POCl_3_ afforded dielectrophile 3-chloro-8-phenyl-6-(thiophen-2-yl)-6,7-dihydro-4*H*-pyrimido [2,1-*c*][1,2,4]triazin-4-one (**9**) with a 75% yield (Scheme 3). The N–H stretching band and its ^1^H-NMR signal for compound **2** disappeared after this step. Hydrazinolysis of compound **9** with an excess of N_2_HNH_2_.H_2_O afforded the hydrazinyltriazine **10**, which undergoes further cyclization due to the reactivity of its hydrazinyl tag, which can be exploited in developing triazolotriazine derivatives. Thus, cyclocondensation of intermediate **10** with phenacyl bromide, triethyl orthoformate, ethyl chloroformate, chloroacetyl chloride, and, finally, dimethylformamide dimethyl acetal (DMF-DMA) afforded the series of compounds **11**, **13**, **14**, and **15** displayed in Scheme 2 under the given conditions. Further hydroxymethylation of compound **11** afforded derivative **12** with a 75% yield. The structure of compound **12** was deduced based on its spectral data, where its mass spectrum recorded a molecular ion peak (C_25_H_20_N_6_O_2_S) at *m*/*z* 468.15, whereas the IR spectrum showed characteristic absorption bands at 3315 cm^−1^ due to stretching of the O–H group. The ^1^H-NMR spectrum displayed a D_2_O exchangeable broad singlet at 5.19 ppm for the O–H group, in addition to a singlet signal at 4.32 ppm that was attributed to the methylene protons of the hydroxymethyl moiety.

**Scheme 4:**
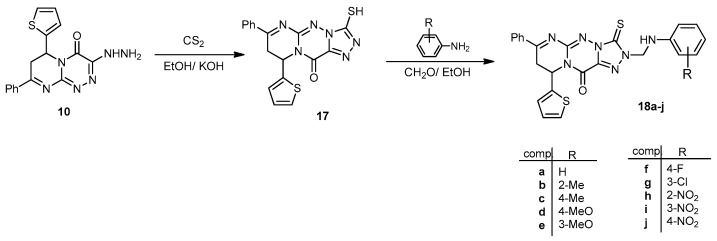
Reagents and conditions for the synthesis of compounds **17** and **18a**–**j**.

Intermediate **10** was cyclocondensed with carbon disulfide to produce the Mannich base precursor **17**, which upon a classical one-pot three-component reaction, produced a set of Mannich bases (**18a**–**j**) with high yields (Scheme 4). The presence of these bases on different pharmacophores have unique potential for medical research [31]. The formation of compounds **18a**–**j** are demonstrated on the basis of the initial Mannich reaction, which proceeds in two steps: First, the reaction between HCHO and the amine leads to the formation of the non-isolable iminium ion intermediate, which loses a H_2_O molecule in situ. Secondly, the thiocarbonyl compound undergoes tautomerization to produce its thiol tautomer, which proceeds to attack the iminium ion, which finally yields the target β-amino-thiocarbonyl compounds (**18a**–**j**) [30]. The IR spectra of the isolated compounds (**18a**–**j**) displayed common characteristic absorption bands around the region 3165–3281 cm^−1^ due to the secondary amine groups. This was further evidenced by their ^1^H-NMR broad singlets at ~4.80 ppm (D_2_O exchangeable), whereas the methylene singlet (^1^H-NMR) of their phenylaminomethyl moiety was observed at ~5.40 ppm. The presence of the nitro group in **18j** was elucidated based on the IR spectrum, which showed two characteristic absorption bands at 1390 and 1520 cm^−1^ due to NO_2*str*_ (*as* and *sym*), respectively. The mass spectrum of **18j** displayed an ion peak at *m*/*z* 530.09 (M^+^, 30%) corresponding to the expected molecular formula C_24_H_18_N_8_O_3_S_2._

**Scheme 5:**
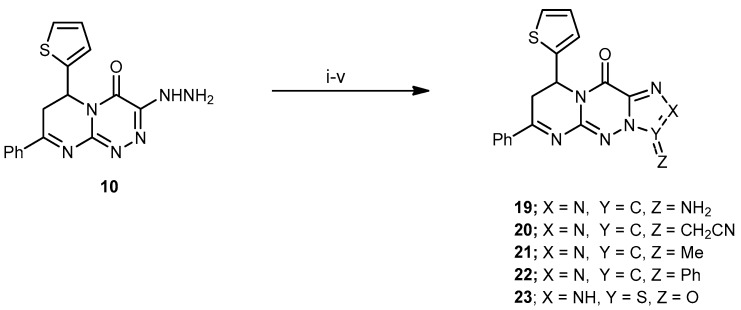
Reagents and conditions for the synthesis of compounds **19**–**23**. Note: i = KSCN/AcOH, ii = CNCH_2_CO_2_Et, iii = Ac_2_O, iv = PhCOCl, and v = SOCl_2_/EtOH.

Upon smooth cyclocondensation of compound **10** with KSCN, ethyl cyanoacetate, acetic anhydride, benzoyl chloride, and thionyl chloride, a series of pyrimido-[1,2,4]triazolo-[1,2,4]triazine derivatives (**19**–**23**) were obtained (Scheme 5). The ^1^H-NMR spectra of these compounds showed the lack of signals corresponding to the hydrazinyl protons originally observed in **10** (^1^H-NMR) at 4.82 and 8.32 ppm. The formation of compound **20** was confirmed through its mass spectrum, which showed a *m*/*z* value at 387.08 corresponding to the expected molecular formula C_19_H_13_N_7_OS, whereas its IR spectrum indicated strong absorption bands at 1686 and 2218 cm^−1^ attributed to the C=O and C≡N groups, respectively.

**Scheme 6:**
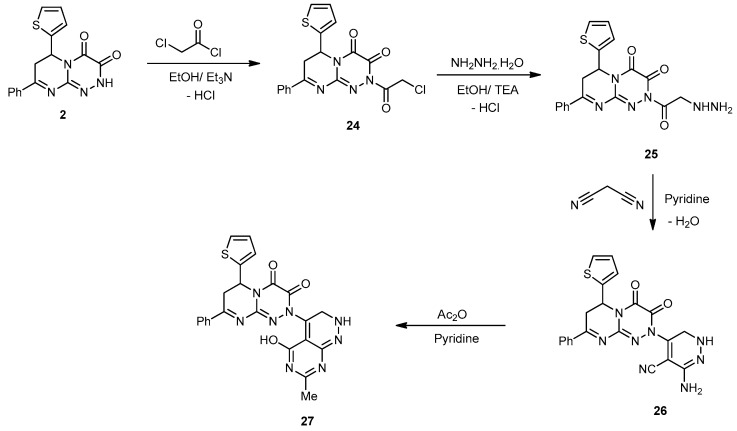
Reagents and conditions for the synthesis of compounds **24**–**27.**

The reactivity of the endocyclic imino moiety in compound **2** was exploited in the annulation of the pendent *N*-(pyridazino-4yl)-pyrimido[2,1-*c*][1,2,4]triazines through consecutive chloroacetylation, hydrazinolysis, and, finally, cyclocondensation with malononitrile. These manipulations afforded compounds **24**–**26**. Further cyclocondensation of compound **26** with acetic anhydride afforded the tetrapod architecture of **27** (Scheme 6). The IR spectrum of **27** revealed the presence of O–H and N–H stretching bands at 3453 and 3251 cm^−1^, respectively. The two amidic C=O groups displayed different stretching bands at 1662 and 1679 cm^−1^, whereas the NH and OH groups showed deuterium-exchangeable signals at ^1^H-NMR broad singlets at 6.92 and 9.31 ppm, respectively. The mass spectrum of **27** displayed an intense peak at *m*/*z* 486.12 (M^+^, 55%) corresponding to the expected molecular formula C_23_H_18_N_8_O_3_S.

**Scheme 7:**
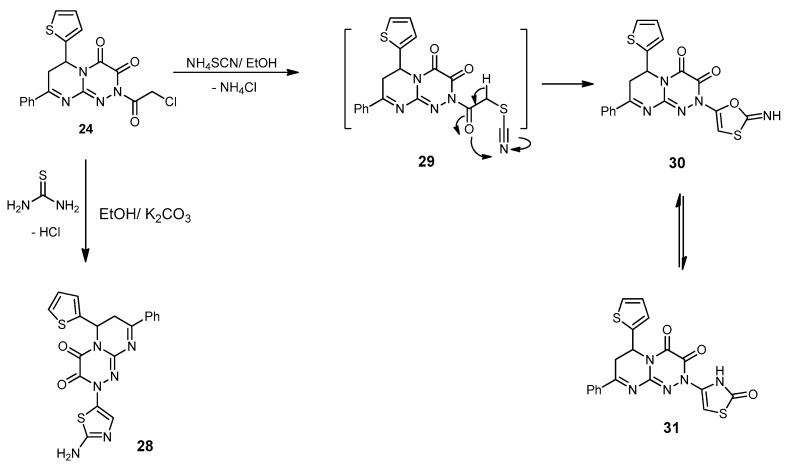
Reagents and conditions for the synthesis of compounds **28** and **31.**

Treatment of compound **24** with thiourea in boiling EtOH/K_2_CO_3_ solution afforded 2-aminothiazole derivative **28** (Scheme 7) but produced thiazolidinone **31** upon a similar reaction with NH_4_SCN in refluxing EtOH. Both reactions proceeded smoothly with good yields. The recorded peak *m*/*z* = 423.03 in addition to the deuterium-exchangeable signal in the ^1^H-NMR spectrum at 10.13 ppm support the structure of compound **31**.

### 2.2. Pharmacological Evaluation

#### Antimicrobial Impact

According to the disc diffusion method [32], compounds **18a**–**j**, **10,** and **17** were screened for their in vitro antimicrobial activity. This series was proposed for antimicrobial screening as it represents the largest homologous series that is suitable for structure–activity relationship (SAR) considerations. The investigations included two Gram-positive strains, *Bacillus subtilis* ATCC 6633 (BS) and *Staphylococcus aureus* ATCC 6535 (SA); two Gram-negative strains, *Pseudomonas aeruginosa* ATCC 27853 (PA) and *Escherichia coli* ATCC 8739 (EC); and two fungal strains, *Candida albicans* ATCC 10231 (CA) and *Aspergillus brasiliensis* ATCC 16404 (AB). Positive controls included ampicillin and gentamicin for Gram-positive and Gram-negative bacteria, respectively, and amphotericin B for fungi, while DMSO was used as the negative control. The minimum inhibitory concentration (MIC) was determined according to the reported method [32].

The inhibitory effects of the synthesized compounds versus these organisms are presented in Table 1. The parent precursors **10** and **17** were less active compared with the triazole’s *N*1-substituted series **18a**–**j**. This finding agrees with the activity of most azole-based antifungal drugs; for instance, fluconazole, ravuconazole, and rufinamide.

Analyses of the MIC values and the inhibition zone diameters, as given in Table 1, show that the test organisms were generally sensitive to compounds **18a**–**j**. The sensitivity ranged from 77% to as high as 100% of the positive controls.

In the case of bacteria, congeners tagged with electron-withdrawing groups **18f**–**j** showed better activity than those modified with electron-donating groups. Electron-withdrawing substituents at the *para* position, as in compounds **18j** and **18f,** displayed higher activity than on the *ortho* or *meta* positions, as in compounds **18i**, **18h,** and **18g**.

This trend deviated for fungi, where derivatives supported by electron-donating groups **18a**–**e** displayed higher activity than compounds **18f**–**j**. Compound **18d**, with a *p*-methoxy tag, was the most potent among those in the tested series. The observed prominent antifungal profile of compounds **18a**–**j** supports our hypothesis that new *N*1-substituted triazole architectures show potential as renewable antifungals. The latent antifungal activity of azoles is attributed to their ability to interfere with and disrupt fungal lanosterol biosynthesis [33], which is required for membrane permeability.

In conclusion, a series of new aza-heterocycles was prepared according to classical chemical methods. They are tripod and tetrapod pharmacophoric architectures that can enhance antimicrobial potency. The derivatives **18a**–**g** displayed promising antimicrobial activities. Derivatives supported by electron withdrawing groups (EWGs) displayed excellent antibacterial activities, whereas those tagged with electron donating groups (EDGs) were better as antifungals. These results support the case for a second phase of biochemical research to elucidate their possible modes of action and determine whether or not these are in line with classical mechanisms.

## 3. Materials and Methods

### 3.1. General Information

Reagents were purchased from Sigma Aldrich (Bayouni Trading Co. Ltd., Al-Khobar, Saudi Arabia) and used without further purification. The reaction progress was monitored by TLC on silica gel pre-coated F254 Merck plates (Merck, Darmstadt, Germany). Spots were visualized by ultraviolet irradiation. All melting points were determined on a digital Gallen-Kamp MFB-595 instrument (Gallenkamp, London, UK) using open capillary tubes and were uncorrected. IR spectra were recorded as potassium bromide discs using Bruker-Vector 22 FTIR spectrophotometer (Bruker, Manasquan, NJ, USA). The NMR spectra were recorded with a Varian Mercury VXR-300 NMR spectrometer (Bruker, Marietta, GA, USA) at 300 and 75 MHz for ^1^H and ^13^C NMR spectra, respectively, using DMSO-*d_6_* as the solvent. Mass spectra were recorded on a Hewlett Packard MS-5988 spectrometer (Hewlett Packard, Palo Alto, CA, USA) at 70 eV. Elemental analyses were conducted at the Micro-Analytical Center of Taif University, Taif, KSA.

The compound 2-Hydrazinyl-6-phenyl-4-(thiophen-2-yl)-4,5-dihydropyrimidine (**1**) was synthesized according to previously reported works [25,26].

### 3.2. Synthesis

*8-Phenyl-6-(thiophen-2-yl)-6,7-dihydro-2H-pyrimido[2,1-c][1,2,4]triazine-3,4-dione* (**2**). A mixture of compound 1 (0.27 g, 1 mmol) and oxalyl chloride (0.12 g, 1 mmol) in DMF (15 mL) containing Et_3_N (0.5 mL) was refluxed for 4 h. After cooling, the reaction mixture was poured onto ice/H_2_O. The formed solid was collected by filtration and recrystallized from EtOH to afford compound **2** as a light yellow powder with a 75% yield; mp 252–254 °C; IR (KBr): (cm^−1^) 689 (C–S–C), 1610–1620 (2 C=N), 1645–1685 (2 amidic C=O), 3235 (N–H); ^1^H-NMR (300 MHz, DMSO-*d_6_*): *δ* 2.82 (dd, *J* = 13.7 and 4.7 Hz, 1H), 2.91 (dd, *J* = 9.5 and 13.7 *Hz*, 1H), 5.09 (dd, *J* = 9.5 and 3.9 *Hz*, 1H), 7.67–7.78 (m, 7H, Ar–H and Thioph._(C3,4)_-2H, 8.05 (d, *J* = 4.5, 1H, Thioph._(C5)_-H), 10.56 (s, 1H, NH _Deutr. Exch_); MS (*m*/*z*, %): 324.07 (M^+^, 10). Anal. Calcd for C_16_H_12_N_4_O_2_S (324.36): C, 59.25; H, 3.73; N, 17.27%. Found: C, 59.02; H, 3.12; N, 17.11%.

*2-(4-Oxo-8-phenyl-6-(thiophen-2-yl)-6,7-dihydro-2H-pyrimido[2,1-c][1,2,4]triazin-3(4H)-ylidene) hydrazinecarbothioamide* (**3**). A mixture of compound **2** (0.32 g, 1 mmol) and thiosemicarbazide (0.09 g, 1 mmol) was refluxed in HCl (0.5 mL) and EtOH (30 mL) for 8 h. The mixture was filtered while hot, and the cooled filtrate was poured onto H_2_O. The formed solid was filtered, washed with H_2_O, and thoroughly dried and recrystallized from EtOH to afford **3** as yellowish-brown powder with an 80% yield; mp 271–273 °C; IR (KBr): (cm^−1^) 685 (C–S–C), 1290 (C=S), 1610–1620 (3 C=N), 1646 (amidic C=O), 3410–3230 (2 NH and NH_2_); ^1^H-NMR (300 MHz, DMSO-*d_6_*): *δ* 2.81 (dd, *J* = 13.7 and 4.7 Hz, 1H), 2.90 (dd, *J* = 9.5 and 13.7 *Hz*, 1H), 5.10 (dd, *J* = 9.5 and 3.9 *Hz*, 1H), 7.67–7.78 (m, 7H, Ar–H and Thioph._(C3,4)_-2H, 8.21 (d, *J* = 4.5, 1H, Thioph._(C5)_-H), 8.56 (s, 2H, NH_2__Deutr. Exch_), 10.59 (s, 1H, NH _Deutr. Exch_); ^13^C-NMR (75 MHz, DMSO-*d_6_*): 41.0 (CH_2_), 51.9 (CH), 127.0, 127.6, 128.1, 128.,2, 128.8, 129.,3, 131.1, 140.5 (C–Ar), 144.6, 149.1, 164.5 (3 C=N–), 160.4 (C=O), 181.2 (C=S); MS (*m*/*z*, %): 397.08 (M^+^, 20). Anal. Calcd for C_17_H_15_N_7_OS_2_ (397.48): C, 51.37; H, 3.80; N, 24.67%. Found: C, 51.10; H, 3.59; N, 24.32%.

*4-Amino-2-((4-oxo-8-phenyl-6-(thiophen-2-yl)-6,7-dihydro-2H-pyrimido[2,1-c][1,2,4]triazin-3(4H)-ylidene)hydrazono)-6-phenyl-2H-1,3-thiazine-5-carbonitrile* (**5**). A mixture of **3** (0.39 g, 1 mmol), and benzylidinemalononitrile (0.15 g, 1 mmol) in dioxane (20 mL) containing a few drops of piperidine (0.3 mL) was refluxed for 6 h. Then, the reaction mixture was left to cool to rt. The solid produced was collected, washed with MeOH, and recrystallized from DMF to produce **5** as yellowish-green solid with a 75% yield; mp 165–167 °C; IR (KBr): (cm^−1^) 685 (C–S–C), 1610–1620 (4 C=N), 1672 (amidic C=O), 2219 (C≡N), 3186–3325 (NH, NH_2_); MS (*m*/*z*, %): 549.12 (M^+^, 18). Anal. Calcd for C_27_H_19_N_9_OS_2_ (549.63): C, 59.00; H, 3.48; N, 22.94%. Found: C, 58.85; H, 3.25; N, 22.67%.

*3-((5-Acetyl-4-methylthiazol-2(3H)-ylidene)hydrazono)-8-phenyl-6-(thiophen-2-yl)-6,7-dihydro-2H-pyrimido[2,1-c][1,2,4]triazin-4(3H)-one* (**6**). 3-Chloro-2,4-pentanedione (0.13 mL, 1 mmol) was added to a solution of compound **3** (0.39 g, 1 mmol) in EtOH (30 mL), and the mixture was refluxed for 6 h. The reaction mixture was cooled down, diluted with H_2_O (50 mL), and sodium acetate (0.49 g, 6 mmol) was added, and the mixture was stirred for 15 min at rt. The obtained product was filtered, washed with H_2_O, dried, and recrystallized from EtOH to produce **6** as a yellow powder with a 65% yield; mp 145–147 °C; IR (KBr): (cm^−1^) 681, 690 (2C–S–C), 1610–1620 (4C=N), 1653, 1710 (2 C=O), 3185–3215 (2 NH); ^1^H-NMR (300 MHz, DMSO-*d_6_*): *δ* 2.51 (s, 3H, Me), 2.72 (s, 3H, MeCO), 2.82 (dd, *J* = 12.4 and 4.6 Hz, 1H), 2.93 (dd, *J* = 8.4 and 12.3 *Hz*, 1H), 5.01 (dd, *J* = 8.6 and 3.8 *Hz*, 1H), 7.68–7.79 (m, 7H, Ar–H and Thioph._(C3,4)_-2H), 8.21 (d, *J* = 4.5, 1H, Thioph._(C5)_-H), 10.21, 10.83 (s, 2H, 2NH_Deutr. Exch_); ^13^C-NMR (75 MHz, DMSO-*d_6_*): 18.4, 25.7 (2Me), 41.0 (CH_2_), 52.3 (CH), 127.1, 127.4, 128.0, 128.2, 128.5, 129.4, 131.1, 140.5 (C–Ar), 144.6, 158.0, 158.3, 164.5 (4 C=N–), 160.3, 192.6 (C=O); MS (*m*/*z*, %): 477.09 (M^+^, 34%). Anal. Calcd for C_22_H_19_N_7_O_2_S_2_ (477.56): C, 55.33; H, 4.01; N, 20.53%. Found: C, 55.19; H, 3.81; N, 20.18%.

*Methyl 2-(4-oxo-2-(-(4-oxo-8-phenyl-6-(thiophen-2-yl)-6,7-dihydro-2H-pyrimido[2,1-c][1,2,4] triazin-3(4H)-ylidene)hydrazono)thiazolidin-5-ylidene)acetate* (**8**). To a well-stirred solution of **3** (0.39 g, 1 mmol) in dry dioxane (20 mL), a solution of dimethyl acetylenedicarboxylate (0.11 mL, 1 mmol) in dioxane (20 mL) was added dropwise. The mixture was stirred for further 2 h at rt, then the reaction mixture was refluxed for a further 8 h (the reaction was monitored by TLC analyses). The solvent was evaporated under a vacuum; the formed solid was filtered and then recrystallized from MeOH–H_2_O to produce **8** as yellowish-brown powder in an 80% yield; mp 228–230 °C; IR (KBr): (cm^−1^) 681, 694 (2C–S–C), 1610–1620 (4C=N), 1653, 1710, 1730 (3 C=O), 3185–3215 (2 NH); ^1^H-NMR (300 MHz, DMSO-*d_6_*): *δ* 2.72 (s, 3H, MeCO), 2.82 (dd, *J* = 12.4 and 4.6 Hz, 1H), 2.93 (dd, *J* = 8.4 and 12.3 *Hz*, 1H), 3.77 (s, 3H, Me), 5.01 (dd, *J* = 8.6 and 3.8 *Hz*, 1H), 6.20 ( s, 1H, =C–H), 7.68–7.79 (m, 7H, Ar–H and Thioph._(C3,4)_-2H), 8.21 (d, *J* = 4.5, 1H, Thioph._(C5)_-H), 10.21, 10.81 (s, 2H, 2NH_Deutr. Exch_); MS (*m*/*z*, %): 507.08 (M^+^, 16%). Anal. Calcd for C_22_H_17_N_7_O_4_S_2_ (507.54): C, 52.06; H, 3.38; N, 19.32%. Found: C, 51.98; H, 3.15; N, 19.18%.

*3-Chloro-8-phenyl-6-(thiophen-2-yl)-6,7-dihydro-4H-pyrimido[2,1-c][1,2,4]triazin-4-one* (**9**) and *3-hydrazinyl-8-phenyl-6-(thiophen-2-yl)-6,7-dihydro-4H-pyrimido[2,1-c][1,2,4]triazin-4-one* (**10**). They were synthesized according to our reported works [25,26].

Compound **9**. Off-white powder with a 75% yield; mp 176–178 °C; IR (KBr): (cm^−1^) 685 (C–Cl), 687 (C–S–C), 1610–1620 (3 C=N), 1645 (amidic C=O); ^1^H-NMR (300 MHz, DMSO-*d_6_*): *δ* 2.83 (dd, *J* = 13.7 and 4.7 Hz, 1H), 2.91 (dd, *J* = 9.5 and 13.7 *Hz*, 1H), 5.14 (dd, *J* = 9.5 and 3.9 *Hz*, 1H), 7.67–7.78 (m, 7H, Ar–H and Thioph._(C3,4)_-2H, 8.25 (d, *J* = 4.5, 1H, Thioph._(C5)_-H); ^13^C-NMR (75 MHz, DMSO-*d_6_*): 41.0 (CH_2_), 51.8 (CH), 127.0, 127.6, 128.1, 128.2, 128.8, 129.3, 131.1, 140.5 (C–Ar), 144.6, 164.5 (2 C=N–), 149.1, (=C–Cl), 160.3 (C=O), 181.2 (C=S); MS (*m*/*z*, %): 342.03 (M^+^, 55), 344.01 (M^+^+2, 18). Anal. Calcd for C_16_H_11_ClN_4_OS (342.80): C, 56.06; H, 3.23; N, 16.34%. Found: C, 55.98; H, 3.01; N, 16.21%.

Compound **10**. Yellowish-brown powder with an 80% yield; mp 220–222 °C; IR (KBr): (cm^−1^) 687 (C–S–C), 1610–1620 (3 C=N), 1645 (amidic C=O), 3211–3351 (NH, NH_2_); ^1^H-NMR (300 MHz, DMSO-*d_6_*): *δ* 2.85 (dd, *J* = 13.7 and 4.7 Hz, 1H), 2.91 (dd, *J* = 9.5 and 13.7 *Hz*, 1H), 4.82 (s, 2H, NH_2Deutr. Exch_), 5.14 (dd, *J* = 9.5 and 3.9 *Hz*, 1H), 7.67–7.78 (m, 7H, Ar–H and Thioph._(C3,4)_-2H, 8.25 (d, *J* = 4.6, 1H, Thioph._(C5)_-H), 8.32 (s, 1H, NH _Deutr. Exch_); MS (*m*/*z*, %): 338.08 (M^+^, 30). Anal. Calcd for C_16_H_14_N_6_OS (338.39): C, 56.79; H, 4.17; N, 24.84%. Found: C, 56.51; H, 4.01; N, 24.63%.

*4,8-Ciphenyl-10-(thiophen-2-yl)-9,10-dihydropyrimido[2,1-c][1,2,4]triazino[3,4-f][1,2,4]triazin-12(2H)-one* (**11**). To a solution of **10** (0.33 g, 1 mmol) in absolute EtOH (25 mL), phenacyl bromide (0.19 g, 1 mmol) was added. The mixture was refluxed for 7 h (monitored by TLC), then left to cool. The precipitate formed was filtered, washed with MeOH, and recrystallized from EtOH to produce **11** as yellow crystals with a 78% yield; mp 242–244 °C; IR (KBr): (cm^−1^) 689 (C–S–C), 1611–1624 (3 C=N), 1648 (amidic C=O), 3231(NH); ^1^H-NMR (300 MHz, DMSO-*d_6_*): *δ* 2.81 (dd, *J* = 13.7 and 4.7 Hz, 1H), 2.90 (dd, *J* = 9.5 and 13.7 *Hz*, 1H), 4.94 (dd, *J* = 9.5 and 3.9 *Hz*, 1H), 7.67–7.78 (m, 13H, Ar–H, Triazin._(C6)_-H and Thioph._(C3,4)_-2H, 8.25 (d, *J* = 4.5, 1H, Thioph._(C5)_-H), 8.32 (s, 1H, NH _Deutr. Exch_); MS (*m*/*z*, %): 438.13 (M^+^, 21). Anal. Calcd for C_16_H_12_N_4_O_2_S (438.50): C, 65.74; H, 4.14; N, 19.17%. Found: C, 65.50; H, 4.07; N, 19.03%.

*2-(Hydroxymethyl)-4,8-diphenyl-10-(thiophen-2-yl)-9,10-dihydropyrimido[2,1-c][1,2,4]triazino [3,4-f][1,2,4]triazin-12(2H)-one* (**12**). A mixture of 11 (0.86 g, 2 mmol) and CH_2_O solution (0.12 mL, 4 mmol) in EtOH (30 mL) was refluxed for 8 h. The precipitate obtained on cooling was filtered, washed several times with H_2_O, and recrystallized from DMF to yield **12** as off-white crystals with a 75% yield; mp 182–184 °C; IR (KBr): (cm^−1^) 685 (C–S–C), 1609–1621(3 C=N), 1645 (amidic C=O), 3315 (OH); ^1^H-NMR (300 MHz, DMSO-*d_6_*): *δ* 2.83 (dd, *J* = 13.7 and 4.7 Hz, 1H), 2.92 (dd, *J* = 9.5 and 13.7 *Hz*, 1H), 4.94 (dd, *J* = 9.5 and 3.9 *Hz*, 1H), 4.32 (s, 2H, CH_2_-OH), 5.19 (s, 1H, OH _Deutr. Exch_), 7.67–7.78 (m, 13H, Ar–H, Triazin._(C6)_-H and Thioph._(C3,4)_-2H), 8.25 (d, *J* = 4.5, 1H, Thioph._(C5)_-H); MS (*m*/*z*, %): 468.15 (M^+^, 60). Anal. Calcd for C_25_H_20_N_6_O_2_S (468.53): C, 64.09; H, 4.30; N, 17.94%. Found: C, 64.11; H, 4.20; N, 17.59%.

*7-Phenyl-9-(thiophen-2-yl)-8H-pyrimido[2,1-c][1,2,4]triazolo[3,4-f][1,2,4]triazin-11(9H)-one* (**13**). Method A: A mixture of compound **10** (0.33 g, 1 mmol) and HC(OEt)_3_ (15 mL) was refluxed for 6 h. The obtained solid on cooling was filtered, dried, and recrystallized from EtOH to yield compound **13** (76%).

Method B: A solution of **10** (0.33 g, 1 mmol) and DMF-DMA (0.22 mL, 2 mmol) in dry xylene (25 mL) was refluxed for 8 h. The solvent was then removed in vacuo, and the solid so obtained was triturated with petroleum ether, collected by filtration, and recrystallized from EtOH to yield **13** as a brown powder with a 62% yield; mp 212–214 °C; IR (KBr): (cm^−1^) 687 (C–S–C), 1610–1620 (4 C=N), 1645 (amidic C=O); ^1^H-NMR (300 MHz, DMSO-*d_6_*): *δ* 2.81 (dd, *J* = 13.5 and 4.5 Hz, 1H), 2.89 (dd, *J* = 9.2 and 13.5 *Hz*, 1H), 5.14 (dd, *J* = 9.5 and 3.9 *Hz*, 1H), 7.67–7.78 (m, 7H, Ar–H and Thioph._(C3,4)_-2H), 8.25 (d, *J* = 4.5, 1H, Thioph._(C5)_-H), 9.52 (s, 1H, Triazol._(C3)_-H); ^13^C-NMR (75 MHz, DMSO-*d_6_*): 32.1, 40.1 (2CH_2_), 51.8 (CH), 127.0, 127.6, 128.1, 128.2, 128.8, 129.3, 131.1, 140.5 (C–Ar), 153.5, 157.6, 160.5, 164.5 (4 C=N–), 160.3 (C=O); MS (*m*/*z*, %): 348.08 (M^+^, 15). Anal. Calcd for C_16_H_12_N_4_O_2_S (348.38): C, 58.61; H, 3.47; N, 24.12%. Found: C, 58.32; H, 3.12; N, 24.01%.

*7-Phenyl-9-(thiophen-2-yl)-8,9-dihydro-2H-pyrimido[2,1-c][1,2,4]triazolo[3,4-f][1,2,4]triazine-3,11-dione* (**14**). To an ice-cooled suspension of KOH (0.11 g, 2 mmol) in absolute EtOH (20 mL), compound **10** (0.66 g, 2 mmol) and ethyl chloroformate (2 mL) were added. The mixture was refluxed for 5 h, then allowed to cool and poured into H_2_O. The precipitate that obtained was filtered and recrystallized from MeOH to afford **14** as brown crystals in a 65% yield; mp 257–259 °C; IR (KBr): (cm^−1^) 687 (C–S–C), 1610–1620 (3 C=N), 1642–1655 (2 amidic C=O), 3210 (NH); ^1^H-NMR (300 MHz, DMSO-*d_6_*): *δ* 2.80 (dd, *J* = 13.7 and 4.7 Hz, 1H), 2.88 (dd, *J* = 9.5 and 13.7 *Hz*, 1H), 5.13 (dd, *J* = 9.5 and 3.9 *Hz*, 1H), 7.65–7.78 (m, 7H, Ar–H and Thioph._(C3,4)_-2H), 8.25 (d, *J* = 4.5, 1H, Thioph._(C5)_-H), 9.41 (s, 1H, NH _Deutr. Exch_); MS (*m*/*z*, %): 364.07 (M^+^, 45). Anal. Calcd for C_17_H_12_N_6_O_2_S (364.38): C, 56.04; H, 3.32; N, 23.06%. Found: C, 56.11; H, 3.03; N, 23.01%.

*3-(Chloromethyl)-7-phenyl-9-(thiophen-2-yl)-8H-pyrimido[2,1-c][1,2,4]triazolo[3,4-f][1,2,4] triazin-11(9H)-one* (**15**). A mixture of **10** (0.33 g, 1 mmol) and chloroacetyl chloride (0.11 mL, 1 mmol) in dry dioxane (25 mL) in the presence of anhydrous K_2_CO_3_ (0.13 g, 1 mmol) was refluxed in a water bath for 8 h. The reaction mixture was evaporated under reduced pressure, and the residue was triturated with H_2_O. The formed solid was filtered off, washed with H_2_O, dried, and recrystallized from EtOH to produce **15** as brown powder with a 76% yield; mp 260–262 °C; IR (KBr): (cm^−1^) 659 (C–Cl), 687 (C–S–C), 1610–1620 (4 C=N), 1645 (amidic C=O); ^1^H-NMR (300 MHz, DMSO-*d_6_*): *δ* 2.82 (dd, *J* = 13.5 and 4.5 Hz, 1H), 2.90 (dd, *J* = 9.2 and 13.5 *Hz*, 1H), 4.23 (s, 2H, CH_2_), 5.16 (dd, *J* = 9.5 and 3.9 *Hz*, 1H), 7.67–7.78 (m, 7H, Ar–H and Thioph._(C3,4)_-2H), 8.20 (d, *J* = 4.5, 1H, Thioph._(C5)_-H); MS (*m*/*z*, %): 398.05 (M^+^+2, 15), 396.02 (42): Anal. Calcd for C_18_H_13_ClN_6_OS (396.85): C, 54.48; H, 3.30; N, 21.18%. Found: C, 54.37; H, 3.11; N, 21.04%.

*3-Mercapto-7-phenyl-9-(thiophen-2-yl)-8H-pyrimido[2,1-c][1,2,4]triazolo[3,4-f][1,2,4]triazin-11(9H)-one* (**17**). To a stirred suspension of compound **10** (0.66 g, 2 mmol) in EtOH (20 mL), ethanolic potassium hydroxide (20 mL, 0.01 mol) and CS_2_ (5 mL) were added dropwise. The reaction mixture was then refluxed for 7 h. After cooling and evaporation of the solvent under reduced pressure, the potassium salt obtained was dissolved in H_2_O and acidified with 2 N aqueous HCl. The precipitate obtained was filtered and recrystallized from EtOH to yield **17** as a colorless powder in a 78% yield; mp 232–234 °C; IR (KBr): (cm^−1^) 687 (C–S–C), 1610–1620 (4 C=N), 1641 (amidic C=O), 2580 (S–H); ^1^H-NMR (300 MHz, DMSO-*d_6_*): *δ* 2.81 (dd, *J* = 13.5 and 4.3 Hz, 1H), 2.90 (dd, *J* = 9.3 and 13.6 *Hz*, 1H), 5.14 (dd, *J* = 9.5 and 3.9 *Hz*, 1H), 7.62–7.78 (m, 7H, Ar–H and Thioph._(C3,4)_-2H), 8.18 (d, *J* = 4.5, 1H, Thioph._(C5)_-H), 11.52 (s, 1H, SH _Deutr. Exch_); ^13^C-NMR (75 MHz, DMSO-*d_6_*): 32.1, 40.1 (2CH_2_), 51.8 (CH), 127.0, 127.6, 128.1, 128.2, 128.8, 129.3, 131.1, 140.5 (C–Ar), 153.5, 157.6, 164.5 (3 C=N–), 160.3 (C=O), 167.9 (N=C–SH); MS (*m*/*z*, %): 380.05 (M^+^, 20). Anal. Calcd for C_17_H_12_N_6_OS_2_ (380.45): C, 53.67; H, 3.18; N, 22.09%. Found: C, 53.38; H, 3.11; N, 22.01%.

General procedure for the synthesis of 2-(((substitutedphenyl)amino)methyl)-7-phenyl-9-(thiophen-2-yl)-3-thioxo-8,9-dihydro-2*H*-pyrimido[2,1-*c*][1,2,4]triazolo[3,4-*f*][1,2,4]triazin-11(3*H*)-ones (**18a**–**j**). A mixture of the thiol compound **17** (0.38 g, 1 mmol); 37% HCHO solution (2 mL); and the selected substituted anilines (1 mmol) in EtOH (30 mL) containing a few drops of HCl was stirred at rt for 7–10 h (monitored by TLC). The obtained solid was collected and recrystallized from the proper solvent to yield the respective products (**18a**–**j**).

*7-Phenyl-2-((phenylamino)methyl)-9-(thiophen-2-yl)-3-thioxo-8,9-dihydro-2H-pyrimido[2,1-c] [1,2,4]triazolo[3,4-f][1,2,4]triazin-11(3H)-one* (**18a**). A yellowish powder from MeOH with a 72% yield; mp 218–220 °C; IR (KBr): (cm^−1^) 687 (C–S–C), 1609–1624 (3 C=N), 1643 (amidic C=O), 1281 C=S), 3219 (N–H); ^1^H-NMR (300 MHz, DMSO-*d_6_*): *δ* 2.79 (dd, *J* = 12.9 and 4.2 Hz, 1H), 2.86 (dd, *J* = 8.8 and 12.9 *Hz*, 1H), 4.82 (s, 1H, N–H _Deutr. Exch_), 5.09 (dd, *J* = 8.9 and 3.8 *Hz*, 1H), 5.42 (s, 2H, CH_2_), 7.68–7.78 (m, 12H, Ar–H and Thioph._(C3,4)_-2H), 8.18 (d, *J* = 4.5, 1H, Thioph._(C5)_-H); ^13^C-NMR (75 MHz, DMSO-*d_6_*): 40.1, 76.1 (2CH_2_), 52.2 (CH), 113.4, 120.8, 127.0, 127.6, 128.1, 128.2, 128.8, 129.3, 129.5, 131.1, 140.6 (C–Ar), 142.6, 144.6, 164.5 (3 C=N–), 160.2 (C=O), 178.9 (C=S); MS (*m*/*z*, %): 485.10 (M^+^, 23). Anal. Calcd for C_24_H_19_N_7_OS_2_ (485.11): C, 59.36; H, 3.94; N, 20.19%. Found: C, 59.14; H, 3.78; N, 20.06%.

*7-Phenyl-9-(thiophen-2-yl)-3-thioxo-2-((o-tolylamino)methyl)-8,9-dihydro-2H-pyrimido[2,1-c] [1,2,4]triazolo[3,4-f][1,2,4]triazin-11(3H)-one* (**18b**). A yellowish powder from acetone with a 79% yield; mp 202–204 °C; IR (KBr): (cm^−1^) 686 (C–S–C), 1610–1625 (3 C=N), 1646 (amidic C=O), 1285 C=S), 3221 (N–H); ^1^H-NMR (300 MHz, DMSO-*d_6_*): *δ* 2.43 (s, 3H, Me), 2.82 (dd, *J* = 13.2 and 4.4 Hz, 1H), 2.87 (dd, *J* = 8.9 and 12.8 *Hz*, 1H), 4.81 (s, 1H, N–H _Deutr. Exch_), 5.10 (dd, *J* = 8.7 and 3.6 *Hz*, 1H), 5.41 (s, 1H, CH_2_), 7.65–7.79 (m, 11H, Ar–H and Thioph._(C3,4)_-2H), 8.20 (d, *J* = 4.5, 1H, Thioph._(C5)_-H); MS (*m*/*z*, %): 499.01 (M^+^, 36). Anal. Calcd for C_25_H_21_N_7_OS_2_ (499.12): C, 60.10; H, 4.24; N, 19.62%. Found: C, 60.01; H, 4.17; N, 19.51%.

*7-Phenyl-9-(thiophen-2-yl)-3-thioxo-4-((o-tolylamino)methyl)-8,9-dihydro-2H-pyrimido[2,1-c] [1,2,4]triazolo[3,4-f][1,2,4]triazin-11(3H)-one* (**18c**). A yellowish powder from MeOH with a 75% yield; mp 165–167 °C; IR (KBr): (cm^−1^) 686 (C–S–C), 1610–1625 (3 C=N), 1646 (amidic C=O), 1285 C=S), 3221 (N–H); ^1^H-NMR (300 MHz, DMSO-*d_6_*): *δ* 2.33 (s, 3H, Me), 2.81 (dd, *J* = 13.2 and 4.4 Hz, 1H), 2.89 (dd, *J* = 8.9 and 12.8 *Hz*, 1H), 4.80 (s, 1H, N–H _Deutr. Exch_), 5.09 (dd, *J* = 8.7 and 3.6 *Hz*, 1H), 5.38 (s, 1H, CH_2_), 7.65–7.79 (m, 11H, Ar–H and Thioph._(C3,4)_-2H), 8.22 (d, *J* = 4.5, 1H, Thioph._(C5)_-H); MS (*m*/*z*, %): 499.02 (M^+^, 51). Anal. Calcd for C_25_H_21_N_7_OS_2_ (499.12): C, 60.10; H, 4.24; N, 19.62%. Found: C, 60.02; H, 4.15; N, 19.49%.

*2-(((4-Methoxyphenyl)amino)methyl)-7-phenyl-9-(thiophen-2-yl)-3-thioxo-8,9-dihydro-2H-pyrimido[2,1-c][1,2,4]triazolo[3,4-f][1,2,4]triazin-11(3H)-one* (**18d**). A yellowish-brown solid EtOH in a 70% yield; mp 196–198 °C; IR (KBr): (cm^−1^) 685 (C–S–C), 1610–1620 (3 C=N), 1643 (amidic C=O), 1291 C=S), 3281 (N–H); ^1^H-NMR (300 MHz, DMSO-*d_6_*): *δ* 2.83 (dd, *J* = 12.8 and 4.1 Hz, 1H), 2.90 (dd, *J* = 8.8 and 12.7 *Hz*, 1H), 3.81 (s, 3H, OMe), 4.81 (s, 1H, N–H _Deutr. Exch_), 5.10 (dd, *J* = 8.7 and 3.6 *Hz*, 1H), 5.40 (s, 1H, CH_2_), 7.60–7.79 (m, 11H, Ar–H and Thioph._(C3,4)_-2H), 8.21 (d, *J* = 4.4, 1H, Thioph._(C5)_-H); MS (*m*/*z*, %): 515.19 (M^+^, 19). Anal. Calcd for C_25_H_21_N_7_O_2_S_2_ (515.61): C, 58.24; H, 4.11; N, 19.02%. Found: C, 58.11; H, 3.98; N, 18.95%.

*2-(((3-Methoxyphenyl)amino)methyl)-7-phenyl-9-(thiophen-2-yl)-3-thioxo-8,9-dihydro-2H-pyrimido[2,1-c][1,2,4]triazolo[3,4-f][1,2,4]triazin-11(3H)-one* (**18e**). Yellow crystals from EtOH with an 80% yield; mp 210–212 °C; IR (KBr): (cm^−1^) 680 (C–S–C), 1613–1624 (3 C=N), 1641 (amidic C=O), 1290 C=S), 3283 (N–H); MS (*m*/*z*, %): 515.13 (M^+^, 23). Anal. Calcd for C_25_H_21_N_7_O_2_S_2_ (515.61): C, 58.24; H, 4.11; N, 19.02%. Found: C, 58.15; H, 3.85; N, 18.98%.

*2-(((3-Fluorophenyl)amino)methyl)-7-phenyl-9-(thiophen-2-yl)-3-thioxo-8,9-dihydro-2H-pyrimido[2,1-c][1,2,4]triazolo[3,4-f][1,2,4]triazin-11(3H)-one* (**18f**). A yellowish powder from acetone in a 75% yield; mp 267–269 °C; IR (KBr): (cm^−1^) 681 (C–S–C), 1610–1620 (3 C=N), 1643 (amidic C=O), 1291 C=S), 3163 (N–H); ^1^H-NMR (300 MHz, DMSO-*d_6_*): *δ* 2.84 (dd, *J* = 12.8 and 4.1 Hz, 1H), 2.94 (dd, *J* = 8.8 and 12.7 *Hz*, 1H), 4.83 (s, 1H, N–H _Deutr. Exch_), 4.09 (dd, *J* = 8.7 and 3.6 *Hz*, 1H), 5.43 (s, 1H, CH_2_), 7.10–8.09 (m, 11H, Ar–H and Thioph._(C3,4)_-2H), 8.20 (d, *J* = 4.4, 1H, Thioph._(C5)_-H); MS (*m*/*z*, %): 503.10 (M^+^, 23). Anal. Calcd for C_24_H_18_FN_7_OS_2_ (503.57): C, 57.24; H, 3.60; N, 19.47%. Found: C, 57.13; H, 3.36; N, 19.25%.

*2-(((3-Chlorophenyl)amino)methyl)-7-phenyl-9-(thiophen-2-yl)-3-thioxo-8,9-dihydro-2H-pyrimido[2,1-c][1,2,4]triazolo[3,4-f][1,2,4]triazin-11(3H)-one* (**18g**). A pale brown powder from DMF–MeOH (1:3) in a 77% yield; mp 276–278 °C; IR (KBr): (cm^−1^) 715 (C–Cl), 681 (C–S–C), 1610–1620 (3 C=N), 1643 (amidic C=O), 1291 C=S), 3163 (N–H); MS (*m*/*z*, %): 522.02 (M^+^ +2, 9), 520.01 (30). Anal. Calcd for C_24_H_18_ClN_7_OS_2_ (520.03): C, 55.43; H, 3.49; N, 18.85%. Found: C, 55.19; H, 3.24; N, 18.63%.

*2-(((2-Nitrophenyl)amino)methyl)-7-phenyl-9-(thiophen-2-yl)-3-thioxo-8,9-dihydro-2H-pyrimido[2,1-c][1,2,4]triazolo[3,4-f][1,2,4]triazin-11(3H)-one* (**18h**). A yellow powder from EtOH in a 71% yield; mp 203–205 °C; IR (KBr): (cm^−1^) 681 (C–S–C), 1610–1620 (3 C=N), 1390, 1520 (NO_2_), 1643 (amidic C=O), 1291 C=S), 3253 (N–H); ^1^H-NMR (300 MHz, DMSO-*d_6_*): *δ* 2.83 (dd, *J* = 12.8 and 4.1 Hz, 1H), 2.90 (dd, *J* = 8.8 and 12.7 *Hz*, 1H), 4.81 (s, 1H, N–H _Deutr. Exch_), 5.10 (dd, *J* = 8.7 and 3.6 *Hz*, 1H), 5.45 (s, 1H, CH_2_), 7.60–8.09 (m, 11H, Ar–H and Thioph._(C3,4)_-2H), 8.21 (d, *J* = 4.4, 1H, Thioph._(C5)_-H); MS (*m*/*z*, %): 530.09 (M^+^, 35). Anal. Calcd for C_24_H_18_ClN_7_OS_2_ (530.58): C, 54.33; H, 3.42; N, 21.12%. Found: C, 54.24; H, 3.19; N, 21.01%.

*2-(((3-Nitrophenyl)amino)methyl)-7-phenyl-9-(thiophen-2-yl)-3-thioxo-8,9-dihydro-2H-pyrimido[2,1-c] [1,2,4]triazolo[3,4-f][1,2,4]triazin-11(3H)-one* (**18i**). A yellow powder from EtOH in a 71% yield; mp 154–456 °C; IR (KBr): (cm^−1^) 681 (C–S–C), 1610–1620 (3 C=N), 1390, 1520 (NO_2_), 1643 (amidic C=O), 1291 C=S), 3253 (N–H); MS (*m*/*z*, %): 530.09 (M^+^, 55). Anal. Calcd for C_24_H_18_ClN_7_OS_2_ (530.58): C, 54.33; H, 3.42; N, 21.12%. Found: C, 54.24; H, 3.19; N, 21.01%.

*2-(((4-Nitrophenyl)amino)methyl)-7-phenyl-9-(thiophen-2-yl)-3-thioxo-8,9-dihydro-2H-pyrimido[2,1-c][1,2,4]triazolo[3,4-f][1,2,4]triazin-11(3H)-one* (**18j**). A yellow powder from MeOH–H_2_O in a 78% yield; mp 281–283 °C; IR (KBr): (cm^−1^) 681 (C–S–C), 1610–1620 (3 C=N), 1390, 1520 (NO_2_), 1643 (amidic C=O), 1291 (C=S), 3253 (N–H); ^1^H-NMR (300 MHz, DMSO-*d_6_*): *δ* 22.83 (dd, *J* = 12.8 and 4.1 Hz, 1H), 2.90 (dd, *J* = 8.8 and 12.7 *Hz*, 1H), 3.81 (s, 3H, OMe), 4.81 (s, 1H, N–H _Deutr. Exch_), 5.10 (dd, *J* = 8.7 and 3.6 *Hz*, 1H), 5.40 (s, 1H, CH_2_), 7.60–7.79 (m, 11H, Ar–H and Thioph._(C3,4)_-2H), 8.21 (d, *J* = 4.4, 1H, Thioph._(C5)_-H); 40.1, 76.1 (2CH_2_), 52.2 (CH), 113.4, 120.8, 127.0, 127.6, 128.1, 128.2, 128.8, 129.3, 129.5, 131.1, 140.6 (C–Ar), 142.6, 144.6, 164.5 (3 C=N–), 160.2 (C=O), 178.9 (C=S); MS (*m*/*z*, %): 530.09 (M^+^, 40). Anal. Calcd for C_24_H_18_ClN_7_OS_2_ (530.58): C, 54.33; H, 3.42; N, 21.12%. Found: C, 54.18; H, 3.25; N, 20.98%.

*3-Amino-7-phenyl-9-(thiophen-2-yl)-8H-pyrimido[2,1-c][1,2,4]triazolo[3,4-f][1,2,4]triazin-11(9H)-one* (**19**). A mixture of compound **10** (0.33 g, 1 mmol) and KSCN (0.48 g, 5 mmol) was refluxed for 7 h in glacial AcOH (25 mL), and the reaction mixture was allowed to cool to rt, then poured into H_2_O. The solid formed was collected by filtration, dried, and recrystallized from EtOH to produce **19** as a light yellow solid in a 76% yield; mp 205–207 °C; IR (KBr): (cm^−1^) 683 (C–S–C), 1613–1625 (4 C=N), 1650 (amidic C=O), 3340 (NH_2_); ^1^H-NMR (300 MHz, DMSO-*d_6_*): *δ* 2.83 (dd, *J* = 13.7 and 4.7 Hz, 1H), 2.91 (dd, *J* = 9.5 and 13.7 *Hz*, 1H), 5.10 (dd, *J* = 9.5 and 3.9 *Hz*, 1H), 6.86 (s, 2H, NH_2__Deutr. Exch_), 7.69–7.78 (m, 7H, Ar–H and Thioph._(C3,4)_-2H, 8.21 (d, *J* = 4.4, 1H, Thioph._(C5)_-H); MS (*m*/*z*, %): 363.09 (M^+^, 50). Anal. Calcd for C_17_H_13_N_7_OS (363.40): C, 56.19; H, 3.61; N, 26.98%. Found: C, 56.01; H, 3.51; N, 26.78%.

*2-(11-Oxo-7-phenyl-9-(thiophen-2-yl)-9,11-dihydro-8H-pyrimido[2,1-c][1,2,4]triazolo[3,4-f] [1,2,4]triazin-3-yl)acetonitrile* (**20**). A mixture of **10** (0.33 g, 1 mmol) and ethyl 2-cyanoacetate (0.11 mL, 1 mmol) in EtOH (30 mL) containing piperidine (0.3 mL) was refluxed for 8 h, then left to cool at rt. The solid product was filtered off, washed with cold EtOH, and recrystallized from EtOH to yield **20** as pale-yellow crystals at a 65% yield; mp 178–180 °C; IR (KBr): (cm^−1^) 687 (C–S–C), 1610–1620 (4 C=N), 1686 (amidic C=O), 2218 (C≡N); ^1^H-NMR (300 MHz, DMSO-*d_6_*): *δ* 2.81 (dd, *J* = 13.5 and 4.5 Hz, 1H), 2.90 (dd, *J* = 9.2 and 13.5 *Hz*, 1H), 3.83 (s, 2H, CH_2_), 5.16 (dd, *J* = 9.5 and 3.9 *Hz*, 1H), 7.67–7.78 (m, 7H, Ar–H and Thioph._(C3,4)_-2H), 8.21 (d, *J* = 4.5, 1H, Thioph._(C5)_-H); ^13^C-NMR (75 MHz, DMSO-*d_6_*): 13.1, 40.1 (2CH_2_), 52.5 (CH), 127.0, 127.6, 128.1, 128.2, 128.8, 129.3, 131.1, 140.5 (C–Ar), 153.5, 157.6, 160.5, 164.5 (4 C=N–), 160.3 (C=O); MS (*m*/*z*, %): 387.08 (M^+^, 75). Anal. Calcd for C_19_H_13_N_7_OS (387.42): C, 58.90; H, 3.38; N, 25.31%. Found: C, 58.71; H, 3.15; N, 25.14%.

*3-Methyl-7-phenyl-9-(thiophen-2-yl)-8H-pyrimido[2,1-c][1,2,4]triazolo[3,4-f][1,2,4]triazin-11(9H)-one* (**21**). Compound **10** (0.33 g, 1 mmol) was dissolved in Ac_2_O (15 mL) and refluxed for 6 h. The reaction mixture was allowed to cool to rt and poured onto H_2_O (50 mL). The resulted solid was filtered off, washed with H_2_O several times, dried, and recrystallized using CHCl_3_ to produce **21** as a brown powder in a 60% yield; mp 156–158 °C; IR (KBr): (cm^−1^) 685 (C–S–C), 1611–1625 (4 C=N), 1645 (amidic C=O); ^1^H-NMR (300 MHz, DMSO-*d_6_*): *δ* 2.43 (s, 3H, Me), 2.81 (dd, *J* = 13.5 and 4.5 Hz, 1H), 2.89 (dd, *J* = 9.2 and 13.5 *Hz*, 1H), 5.14 (dd, *J* = 9.5 and 3.9 *Hz*, 1H), 7.67–7.78 (m, 7H, Ar–H and Thioph._(C3,4)_-2H), 8.25 (d, *J* = 4.5, 1H, Thioph._(C5)_-H); MS (*m*/*z*, %): 362.09 (M^+^, 42). Anal. Calcd for C_18_H_14_N_6_OS (362.41): C, 59.65; H, 3.89; N, 23.19%. Found: C, 59.47; H, 3.62; N, 23.07%.

*3,7-Diphenyl-9-(thiophen-2-yl)-8H-pyrimido[2,1-c][1,2,4]triazolo[3,4-f][1,2,4]triazin-11(9H)-one* (**22**). A mixture of compound **10** (0.33 g, 1 mmol, and benzoyl chloride (15 mL) was refluxed for 4 h. The reaction mixture was allowed to cool to rt; the resulting solid was filtered off, washed with cold MeOH, dried, and recrystallized using MeOH to yield **22** as orange crystals at a 76% yield; mp 275–277 °C; IR (KBr): (cm^−1^) 680 (C–S–C), 1614–1630 (4 C=N), 1641 (amidic C=O); ^1^H-NMR (300 MHz, DMSO-*d_6_*): *δ* 2.45 (s, 3H, Me), 2.83 (dd, *J* = 13.5 and 4.5 Hz, 1H), 2.92 (dd, *J* = 9.2 and 13.5 *Hz*, 1H), 5.16 (dd, *J* = 9.5 and 3.9 *Hz*, 1H), 7.67–7.78 (m, 12H, Ar–H and Thioph._(C3,4)_-2H), 8.22 (d, *J* = 4.5, 1H, Thioph._(C5)_-H); MS (*m*/*z*, %): 424.11 (M^+^, 50). Anal. Calcd for C_16_H_12_N_4_O_2_S (424.48): C, 65.08; H, 3.80; N, 19.80%. Found: C, 65.01; H, 3.71; N, 19.51%.

*7-Phenyl-9-(thiophen-2-yl)-8,9-dihydropyrimido[2,1-c][1,2,3,5]thiatriazolo[4,5-f][1,2,4]triazin-11 (2H)-one 3-oxide* (**23**). A solution of compound **10** (0.33 g, 1 mmol) in EtOH (25 mL) treated with EtONa (0.23 g, 10 mmol sodium metal in 5 mL absolute EtOH) and thionyl chloride was heated in a water bath for 8 h. The mixture was diluted with H_2_O and extracted with ethyl acetate; the organic layers were dried over anhydrous Na_2_SO_4_; the ethyl acetate was removed under reduced pressure and recrystallized by EtOH to produce **23** as a yellow solid in a 65% yield; mp 231–233 °C; IR (KBr): (cm^−1^) 685 (C–S–C), 1611–1625 (4 C=N), 1331 (S=O), 1645 (amidic C=O); ^1^H-NMR (300 MHz, DMSO-*d_6_*): *δ* 2.80 (dd, *J* = 13.5 and 4.5 Hz, 1H), 2.90 (dd, *J* = 9.2 and 13.5 *Hz*, 1H), 5.11 (dd, *J* = 9.5 and 3.9 *Hz*, 1H), 7.65–7.79 (m, 7H, Ar–H and Thioph._(C3,4)_-2H), 10.01 (s, 1H, NH _Deutr. Exch_); MS (*m*/*z*, %): 384.05 (M^+^, 10). Anal. Calcd for C_16_H_12_N_6_O_2_S_2_ (384.44): C, 49.99; H, 3.15; N, 21.86%. Found: C, 49.78; H, 3.02; N, 21.75%.

*2-(2-Chloroacetyl)-8-phenyl-6-(thiophen-2-yl)-6,7-dihydro-2H-pyrimido[2,1-c][1,2,4]triazine-3,4-dione* (**24**). A mixture of chloroacetyl chloride (0.02 mol) in absolute EtOH (20 mL) was added dropwise during 30 min to a well-stirred solution of **2** (0.32 g, 1 mmol) in absolute EtOH (30 mL) containing Et_3_N (0.5 mL). The mixture was stirred for a further 1 h at 80 °C; the reaction mixture was allowed to cool to rt, and then poured onto ice-cooled H_2_O. The produced solid was filtered off, dried, and recrystallized from EtOH/dioxane (2:1) to produce **24** as a yellowish-green powder in an 80% yield; mp 128–130 °C; IR (KBr): (cm^−1^) 688 (C–S–C), 715 (C–Cl), 1610–1626 (2 C=N), 1641–1682 (3 amidic C=O); ^1^H-NMR (300 MHz, DMSO-*d_6_*): *δ* 2.82 (dd, *J* = 13.7 and 4.7 Hz, 1H), 2.91 (dd, *J* = 9.5 and 13.8 *Hz*, 1H), 4.21 (s, 2H, CH_2_), 5.10 (dd, *J* = 9.5 and 3.9 *Hz*, 1H), 7.67–7.78 (m, 7H, Ar–H and Thioph._(C3,4)_-2H, 8.04 (d, *J* = 4.5, 1H, Thioph_.(C5)_-H); ^13^C-NMR (75 MHz, DMSO-*d_6_*): 41.0, 42.4 (2CH_2_), 51.8 (CH), 127.1, 127.4, 128.0, 128.2, 128.5, 129.4, 131.1, 140.5 (C–Ar), 144.6, 164.5 (2 C=N–), 157.1, 159.0, 165.7 (3C=O); MS (*m*/*z*, %): 402.05 (M^+^+2, 20), 400.04 (58). Anal. Calcd for C_18_H_13_ClN_4_O_3_S (400.84): C, 53.94; H, 3.27; N, 13.98%. Found: C, 53.76; H, 3.11; N, 13.75%.

*2-(2-Hydrazinylacetyl)-8-phenyl-6-(thiophen-2-yl)-6,7-dihydro-2H-pyrimido[2,1-c]*[1,2,4]*triazine-3,4-dione* (**25**). It was prepared according to the general procedure described for compound **10** as a reddish-brown powder from EtOH in a 76% yield; mp 227–229 °C; IR (KBr): (cm^−1^) 686 (C–S–C), 1610–1626 (2 C=N), 1641–1682 (3 amidic C=O), 3186–3350 (NH and NH_2_); ^1^H-NMR (300 MHz, DMSO-*d_6_*): *δ* 2.79 (dd, *J* = 13.7 and 4.7 Hz, 1H), 2.86 (dd, *J* = 9.5 and 13.8 *Hz*, 1H), 3.12 (s, 2H, CH_2_), 4.50 (s, 2H, NH_2__Deutr. Exch_), 5.10 (dd, *J* = 9.5 and 3.9 *Hz*, 1H), 7.67–7.78 (m, 7H, Ar–H and Thioph._(C3,4)_-2H, 8.04 (d, *J* = 4.5, 1H, Thioph._(C5)_-H), 8.31 (s, 1H, NH_Deutr. Exch_); MS (*m*/*z*, %): 396.10 (M^+^, 15). Anal. Calcd for C_18_H_16_N_6_O_3_S (396.42): C, 54.54; H, 4.07; N, 21.20%. Found: C, 54.36; H, 3.87; N, 21.11%.

*3-Amino-5-(3,4-dioxo-8-phenyl-6-(thiophen-2-yl)-3,4,6,7-tetrahydro-2H-pyrimido[2,1-c][1,2,4] triazin-2-yl)–1,6-dihydropyridazine-4-carbonitrile* (**26**). To a solution of **25** (0.78 g, 1 mmol) in pyridine (15 mL), malononitrile (0.12 g, 2 mmol) was added, and the mixture was refluxed for 6 h. The reaction mixture was left to cool at rt. The precipitate that formed was collected and recrystallized from CHCl_3_ to yield **26** as yellow-orange powder in a 75% yield; mp 221–223 °C; IR (KBr): (cm^−1^) 685 (C–S–C), 1610–1620 (3 C=N), 1665–1686 (2amidic C=O), 2218 (C≡N), 3186–3350 (NH and NH_2_); ^1^H-NMR (300 MHz, DMSO-*d_6_*): *δ* 2.83 (dd, *J* = 13.5 and 4.5 Hz, 1H), 2.94 (dd, *J* = 9.2 and 13.5 *Hz*, 1H), 3.34 (s, 2H, Pyridaz._(C6)_-2H), 5.12 (dd, *J* = 9.5 and 3.9 *Hz*, 1H), 6.81 (s, H, NH_Deutr. Exch_), 7.67–7.78 (m, 7H, Ar–H and Thioph._(C3,4)_-2H), 8.21 (d, *J* = 4.5, 1H, Thioph._(C5)_-H), 8.50 (s, 2H, NH_2__Deutr. Exch_); MS (*m*/*z*, %): 444.10 (M^+^, 62). Anal. Calcd for C_21_H_16_N_8_O_2_S (444.47): C, 56.75; H, 3.63; N, 25.21%. Found: C, 56.54; H, 3.41; N, 25.12%.

*2-(5-Hydroxy-7-methyl-2,3-dihydropyrimido[4,5-c]pyridazin-4-yl)-8-phenyl-6-(thiophen-2-yl)-6,7-dihydro-2H-pyrimido[2,1-c][1,2,4]triazine-3,4-dione* (**27**). A solution of **26** (0.44 g, 1 mmol) in an Ac_2_O/pyridine mixture (30 mL, (2:1)) was heated in a water bath for 8 h, then cooled and poured into an ice/H_2_O mixture. The precipitate thus obtained was filtered off, washed several times with H_2_O, dried, and recrystallized from EtOH to yield **27** as yellowish crystals in a 76% yield; mp 240–242 °C; IR (KBr): (cm^−1^) 685 (C–S–C), 1615–1630 (4 C=N), 1662, 1679 (2amidic C=O), 3251 (NH), 3453 (br OH); ^1^H-NMR (300 MHz, DMSO-*d_6_*): *δ* 1.04 (s, 3H, Me), 2.79 (dd, *J* = 13.5 and 4.5 Hz, 1H), 2.87 (dd, *J* = 9.2 and 13.5 *Hz*, 1H), 3.32 (s, 2H, Pyridaz._(C6)_-2H), 5.10 (dd, *J* = 9.5 and 3.9 *Hz*, 1H), 6.92 (s, H, NH_Deutr. Exch_); 7.67–7.78 (m, 7H, Ar–H and Thioph._(C3,4)_-2H), 8.21 (d, *J* = 4.5, 1H, Thioph._(C5)_-H), 9.31 (s, 1H, OH_Deutr. Exch_); MS (*m*/*z*, %): 486.12 (M^+^, 55%). Anal. Calcd for C_23_H_18_N_8_O_3_S (486.51): C, 56.78; H, 3.73; N, 23.03%. Found: C, 56.50; H, 3.61; N, 23.01%.

*2-(2-Aminothiazol-5-yl)-8-phenyl-6-(thiophen-2-yl)-6,7-dihydro-2H-pyrimido[2,1-c][1,2,4] triazine-3,4-dione* (**28**). A mixture of **24** (0.40 g, 1 mmol) and an equimolar amount (1 mmol) of thiourea in dry EtOH (30 mL) containing anhydrous K_2_CO_3_ (0.13 g, 1 mmol) was refluxed for 8 h. The excess solvent was removed under reduced pressure, and the residue was triturated with MeOH. The formed product was filtered, washed with cold MeOH, dried, and recrystallized from the DMF–MeOH (1:3) to yield **28** as a brown solid in an 80% yield; mp 231–233 °C; IR (KBr): (cm^−1^) 679, 685 (2C–S–C), 1610–1620 (3 C=N), 1665–1686 (2amidic C=O), 3215 (NH_2_); ^1^H-NMR (300 MHz, DMSO-*d_6_*): *δ* 2.78 (dd, *J* = 12.4 and 4.6 Hz, 1H), 2.91 (dd, *J* = 8.1 and 12.1 *Hz*, 1H), 5.01 (dd, *J* = 8.6 and 3.8 *Hz*, 1H), 6.21 (s, 1H, Thiaz._(C4)_-H), 6.51 (s, 2H, NH_2__Deutr. Exch_), 7.68–7.79 (m, 7H, Ar–H and Thioph._(C3,4)_-2H), 8.21 (d, *J* = 4.5, 1H, Thioph._(C5)_-H); MS (*m*/*z*, %): 422.06 (M^+^, 20). Anal. Calcd for C_19_H_14_N_6_O_2_S_2_ (422.48): C, 54.01; H, 3.34; N, 19.89%. Found: C, 54.12; H, 3.11; N, 19.57%.

*2-(2-Oxo-2,3-dihydrothiazol-4-yl)-8-phenyl-6-(thiophen-2-yl)-6,7-dihydro-2H-pyrimido[2,1-c][1,2,4]triazine-3,4-dione* (**31**). A mixture of **24** (0.40 g, 1 mmol) and NH_4_SCN (0.15 g, 2 mmol) in absolute EtOH (30 mL) was refluxed for 8 h. The reaction mixture was allowed to stand overnight; the solid produced was filtered and then recrystallized from EtOH to produce **31** as yellow crystals in a 76% yield; mp 189–191 °C; IR (KBr): (cm^−1^) 679, 689 (2C–S–C), 1610–1620 (C=N), 1651–1686 (3 amidic C=O), 3185 (NH); ^1^H-NMR (300 MHz, DMSO-*d_6_*): *δ* 2.78 (dd, *J* = 12.4 and 4.6 Hz, 1H), 2.92 (dd, *J* = 8.4 and 12.3 *Hz*, 1H), 5.01 (dd, *J* = 8.6 and 3.8 *Hz*, 1H), 6.83 (s, 1H, Thiaz._(C5)_-H), 7.68–7.79 (m, 7H, Ar–H and Thioph._(C3,4)_-2H), 8.21 (d, *J* = 4.5, 1H, Thioph._(C5)_-H), 10.13 (s, 1H, NH_Deutr. Exch_); MS (*m*/*z*, %): 423.03 (M^+^, 75). Anal. Calcd for C_19_H_13_N_5_O_3_S_2_ (423.47): C, 53.89; H, 3.09; N, 16.54%. Found: C, 53.68; H, 3.21; N, 16.28%.

### 3.3. Antimicrobial Assessment

#### Methodology

The antimicrobial activity of the new synthesized compounds was evaluated using the disc diffusion method [32]. Plates 90 mm in diameter containing either Müller–Hinton agar for the growth of bacteria or Sabouraud dextrose agar for the growth of fungi were prepared, and each plate was separately inoculated with different cultures of the test bacteria and fungi by aseptically swabbing onto the entire surface of the agar with cotton wool. A 6-mm-diameter filter paper disc was saturated with 200 μg/mL of the test compound in DMSO. The discs were air-dried and placed aseptically at the center of the plates. The plates were left in a refrigerator for 1 h before incubation to allow the extract to diffuse into the agar. Ampicillin and gentamicin were used as bacterial standards and amphotericin B as the fungal reference to evaluate the efficacy of the tested compounds, with DMSO used as a negative control. After incubation of the plate at a suitable temperature (37 °C for bacteria and 25 °C for fungi), the results were recorded for each tested compound as the average diameter (mm) of the inhibition zone (IZ) of bacterial or fungal growth around the discs. The minimum inhibitory concentration (MIC) was determined for compounds that exhibited significant growth inhibition zones of more than 15 mm using the two-fold serial dilution method [34]. The MIC (µM) and IZ values are listed in Table 1.

## 4. Conclusions

In conclusion, a novel series of annelated pyrimido[2,1-*c*][1,2,4]triazolo[3,4-*f*][1,2,4]triazines were synthesized and evaluated for their in vitro antimicrobial activity. We synthesized 8-phenyl-6-(thiophen-2-yl)-6,7-dihydro-2*H*-pyrimido[2,1-*c*][1,2,4]triazine-3,4-dione (**2**), 2-(4-oxo-8-phenyl-6-(thiophen-2-yl)-6,7-dihydro-2*H*-pyrimido[2,1-*c*][1,2,4]triazin-3(4*H*)-ylidene)hydrazinecarbothioamide (**3**), and 3-hydrazinyl-8-phenyl-6-(thiophen-2-yl)-6,7-dihydro-4*H*-pyrimido[2,1-*c*][1,2,4]triazin-4-one (**10**) as new two-pharmacophoric-motif precursors in considerable yields. The reactivity of the hydrazinyl tag and thioureido moieties were investigated in an assortment of manipulations involving cyclocondensation, aiming at annulation of the new three-pharmacophoric-motif conjugates. The results of antimicrobial screening showed that conjugates **18j**, **18f**, **18i**, **18h**, **18g**, **18a**, **18b**, **18c**, **18e**, and **18d** exhibited significant antimicrobial activities in decreasing order of listing, whereas compounds **10** and **17** showed less potency. Compounds with an electron-withdrawing moiety—for instance, NO_2_ and fluro at the *para* and *meta* positions—exhibited the most significant antibacterial activity. Notably, the *para* position was more convenient for augmenting the antibacterial activity. Compound **18d**, with an electron-releasing group at the *para* position, was found to be the most powerful antifungal analogue, with MIC values 27.77 and 27.44 µM, in hindering the growth of AB and CA, respectively.

## Figures and Tables

**Table 1:** Preliminary antimicrobial activity for some new synthesized compounds.

Compound No.	MIC Values, μM ^a^ (Inhibition Zone (mm))					
	Gram-Positive Bacteria		Gram-Negative Bacteria		Fungi	
	SA	BS	PA	EC	AB	CA
**10**	107.12 (25)	110.97 (25)	118.00 (25)	113.04 (25)	134.02 (20)	127.69 (23)
**17**	85.29 (24)	86.5 (24)	92.02 (24)	91.26 (25)	99.49 (23)	96.73 (23)
**18a**	34.67 (30)	35.06 (29)	37.43 (29)	36.73 (30)	68.52 (24)	68.34 (25)
**18b**	38.07 (29)	39.11 (29)	41.31 (28)	40.37 (29)	34.56 (30)	32.40 (30)
**18c**	45.32 (28)	45.78 (28)	48.59 (29)	46.70 (30)	29.35 (32)	29.21 (33)
**18d**	59.77 (26)	60.34 (25)	65.65 (26)	62.28 (26)	27.77 (33)	27.44 (33)
**18e**	52.48 (27)	54.60 (26)	58.20 (28)	56.86 (27)	29.40 (31)	29.25 (32)
**18f**	22.47 (34)	22.57 (33)	25.24 (33)	24.86 (32)	37.83 (28)	37.43 (29)
**18g**	27.21 (31)	26.31 (31)	32.94 (30)	31.21 (31)	58.09 (24)	57.79 (25)
**18h**	24.58 (32)	25.10 (31)	27.40 (31)	26.42 (32)	45.52 (25)	45.35 (26)
**18i**	22.81 (33)	22.92 (32)	25.16 (32)	24.93 (32)	39.62 (27)	37.88 (28)
**18j**	21.34 (34)	21.37 (34)	23.01 (33)	22.71 (33)	34.60 (29)	34.47 (28)
Control^*^	20.21 (35)	20.23 (34)	22.27 (33)	22.07 (35)	15.30 (33)	14.53 (34)
DMSO	-	-	-	-	-	-

^a^ Values are the average of three readings. Minimum inhibitory concentrations (MICs): more than 59 μM, slight activity; 34–59 μM, moderate activity; and 21–35 μM, high activity. * Ampicillin in the case of Gram-positive bacteria, gentamicin in the case of Gram-negative bacteria, and amphotericin B in the case of fungi.

## References

[1] Jaiswal S., Singh D.K., Shukla P. (2019). Gene editing and systems biology tools for pesticide bioremediation: A review. Front. Microbiol..

[2] Bush K. (2018). Past and present perspectives on -lactamases. Antimicrob. Agents Chemother.

[3] Lumactud R., Fulthorpe R.R. (2018). Endophytic bacterial community structure and function of herbaceous plants from petroleum hydrocarbon contaminated and non-contaminated sites. Front. Microbiol..

[4] Fitzgerald D.M., Rosenberg S.M. (2019). What is mutation? a chapter in the series: How microbes “jeopardize” the modern synthesis. PLoS Genetics.

[5] Moore J.M., Correa R., Rosenberg S.M., Hastings P.J. (2017). Persistent damaged bases in DNA allow mutagenic break repair in *Escherichia coli*. PLoS Genet.

[6] Goh Y.J., Barrangou R. (2019). Harnessing CRISPR-Cas systems for precision engineering of designer probiotic lactobacilli. Curr. Opin. Biotechnol..

[7] Arciszewska K., Pućkowska A., Wróbel A., Drozdowska D. (2019). Carbocyclic analogues of distamycin and netropsin. Mini Rev. Med. Chem..

[8] Tang X., Shijun S., Chen M., Jun H., Rongjiao X., Tao G., Chen Y., Zhang C., Wang J., Min Y. (2019). Novel chalcone derivatives containing a 1,2,4-triazine moiety: Design, synthesis, antibacterial and antiviral activities. RSC Adva..

[9] František Z., Slouka J., Stýskala J. (2019). General Synthesis of 1-Aryl-6-azaisocytosines and their utilization for the preparation of related condensed 1,2,4-triazines. Molecules.

[10] Abou-Elregal M.K., Mohamed A.T.A., Youssef A.S.A., Hemdan M.M., Samir S.S., Abou-Elmagd W.S.I. (2018). Synthesis and antitumor activity evaluation of some 1,2,4-triazine and fused triazine derivatives. Synth. Commun..

[11] Abdallah A.E.M., Elgemeie G.H. (2018). Design, synthesis, docking, and antimicrobial evaluation of some novel pyrazolo[1,5-*a*] pyrimidines and their corresponding cycloalkane ring-fused derivatives as purine analogs. Drug. Des. Devel. Ther..

[12] Havránková E., Csöllei J., Pazdera P. (2019). New approach for the one-pot synthesis of 1,3,5-triazine derivatives: application of Cu(I) supported on a weakly acidic cation-exchanger resin in a comparative Study. Molecules.

[13] Supuran C. (2018). Carbonic Anhydrases and metabolism. Metabolites.

[14] Alrawashdeh M.S.M. (2018). Determination of antimicrobial activity of some 1,2,4-triazole derivatives. Regul. Mech. Biosyst..

[15] Wenjing Y., Qi Y., Simiao Y., Ping G., Mingze Q. (2017). Synthesis and antitumor activity of triazole-containing sorafenib analogs. Molecules.

[16] El-Sherief H.A.M., Youssif B.G.M., Abbas Bukhari S.N., Abdelazeem A.H., Abdel-Aziz M., Abdel-Rahman H.M. (2018). Synthesis, anticancer activity and molecular modeling studies of 1,2,4-triazole derivatives as EGFR inhibitors. Eur. J. Med. Chem..

[17] Lakkakulaa R., Roya A., Mukkantib K., ridharc G.S. (2019). Synthesis and anticancer activity of 1,2,3-triazole fused *N*-arylpyrazole derivatives. Russ. J. Gen. Chem..

[18] Prakash B.K., Karabasanagoud T., Ramesh S.G. (2019). Synthesis, characterization and antimicrobial activity of some novel Schiff and Mannich bases derived from 1,2,4- triazoles. Int. Res. J. Pharm..

[19] Tariq S., Kamboj P., Alam O., Amir M. (2018). 1,2,4-Triazole-based benzothiazole/benzoxazole derivatives: Design, synthesis, p38α MAP kinase inhibition, anti-inflammatory activity and molecular docking studies. Bioorg. Chem..

[20] Chunyan L., Min B., Lijun Y., Chengxi W. (2018). Synthesis and anti-inflammatory activity evaluation of 5-(1-benzyl–1*H*-[1,2,3]triazol-4-yl)-4-phenyl-4*H*-[1,2,4]triazole-3-thiol derivatives. Ind. J. Pharma. Educ. Res..

[21] Ibrahim U., Gokalp-Ozkorkmaz E., Engin D. (2019). Experimentally induced diabetes mellitus influences expression of VEGF and CD68 in rat teeth pulp. Int. J. Morphol..

[22] Osasenaga M.I., Abiola M.A., Oluseyi A.A. (2017). Alloxan-induced diabetes, a common model for evaluating the glycemic-control potential of therapeutic compounds and plants extracts in experimental studies. Medicina.

[23] Yin P., Wang Y., Yang L., Sui J., Liu Y. (2018). Hypoglycemic effects in alloxan-induced diabetic rats of the phenolic extract from mongolian oak cups enriched in ellagic acid, kaempferol and their derivatives. Molecules.

[24] El Azab I.H., Elkanzy N.A.A. (2017). Synthesis and anti-tumour activity of some new sebacoyl chloride-based heterocycles. Curr. Org. Synth..

[25] El Azab I.H., Aly M.R.E., Gobouri A.A. (2017). Synthesis of new azole and azine systems based on chromeno[3,4-*c*]pyrrole-3,4-dione and investigation of their cytotoxic activity. Heterocycles.

[26] El Azab I.H., Elkanzy N.A.A., Gobouri A.A. (2018). Design and synthesis of some new quinoxaline-based heterocycles. J. Heterocycl. Chem..

[27] El Azab I.H., Gobouri A.A., Altalhi T.A. (2019). 4-Chlorothiazole-5-carbaldehydes as potent precursors for synthesis of some new pendant *N*-heterocyces endowed with anti-tumor activity. J. Heterocycl. Chem..

[28] El Azab I.H., Elkanzy N.A.A., Gobouri A.A., Altalhi T.A. (2019). Convenient synthesis of novel nitrogen bridgehead heterocycles utilizing 3-mercapto-6*H*-[1,2,4,5]oxatriazino[3,2-*a*] isoindol-6-one as a new synthon. J. Heterocycl. Chem..

[29] El Azab I.H., Abu Ali O.A., El-Zahrani A.H., Gobouri A.A., Altalhi T.A. (2019). Pyrazole-1-carbothioamide as a potent precursor for synthesis of some new *N*-heterocycles of potential biological activity. J. Heterocycl. Chem..

[30] El Azab I.H., Khalifa M.E., Gobouri A.A., Altalhi T.A. (2019). Synthesis, sharacterization, and pharmacological evaluation of some new pteridine-based heterocycles as antimicrobial agents. J. Heterocycl. Chem..

[31] Łukasz P., Jolanta R., Urszula K., Anna H., Monika W., Anna M. (2014). Synthesis, antiproliferative and antimicrobial activity of new Mannich bases bearing 1,2,4-triazole moiety. J. Enzyme Inhib. Med. Chem..

[32] Bondock S., Rabie R., Etman H.A., Fadda A.A. (2008). Synthesis and antimicrobial activity of some new heterocycles incorporating antipyrine moiety. Eur. J. Med. Chem..

[33] Abuhammad A. (2017). Cholesterol metabolism: a potential therapeutic target in *Mycobacteria* Br. J. Pharmacol..

[34] Chung P.Y., Chung L.Y., Ngeow Y.F., Goh S.H., Imiyabir Z. (2004). Antimicrobial activities of malaysian plant species. Pharm. Biol..

